# Myoplasmic resting Ca^2+^ regulation by ryanodine receptors is under the control of a novel Ca^2+^-binding region of the receptor

**DOI:** 10.1042/BJ20131553

**Published:** 2014-05-13

**Authors:** Yanyi Chen, Shenghui Xue, Juan Zou, Jose R. Lopez, Jenny J. Yang, Claudio F. Perez

**Affiliations:** *Department of Chemistry, Center for Diagnostics and Therapeutics, Georgia State University, 50 Decatur Street, NSC 552, Atlanta, GA 30303, U.S.A.; †Department of Molecular Biosciences, School of Veterinary Medicine, University of California, Davis, 1089 Veterinary Medicine Drive, Davis, CA 95616, U.S.A.; ‡Department of Anesthesiology, Perioperative and Pain Medicine, Brigham and Women's Hospital, Harvard Medical School, 20 Shattuck Street, Boston, MA 02115, U.S.A.

**Keywords:** calcium-binding site, calcium leak, myotube, skeletal muscle, terbium fluorescence, tryptophan fluorescence, [Ca^2+^]_rest_, intracellular resting myoplasmic free Ca^2+^ concentration, CLR, Ca^2+^ leak regulatory, DHPR, dihydropyridine receptor, fura 2/AM, fura 2 acetoxymethyl ester, HEK, human embryonic kidney, MHS, malignant hyperthermia syndrome, RyR, ryanodine receptor, SERCA1, sarcoplasmic/endoplasmic reticulum Ca^2+^-ATPase 1, SR, sarcoplasmic reticulum

## Abstract

Passive SR (sarcoplasmic reticulum) Ca^2+^ leak through the RyR (ryanodine receptor) plays a critical role in the mechanisms that regulate [Ca^2+^]_rest_ (intracellular resting myoplasmic free Ca^2+^ concentration) in muscle. This process appears to be isoform-specific as expression of either RyR1 or RyR3 confers on myotubes different [Ca^2+^]_rest_. Using chimaeric RyR3–RyR1 receptors expressed in dyspedic myotubes, we show that isoform-dependent regulation of [Ca^2+^]_rest_ is primarily defined by a small region of the receptor encompassing amino acids 3770–4007 of RyR1 (amino acids 3620–3859 of RyR3) named as the CLR (Ca^2+^ leak regulatory) region. [Ca^2+^]_rest_ regulation by the CLR region was associated with alteration of RyRs’ Ca^2+^-activation profile and changes in SR Ca^2+^-leak rates. Biochemical analysis using Tb^3+^-binding assays and intrinsic tryptophan fluorescence spectroscopy of purified CLR domains revealed that this determinant of RyRs holds a novel Ca^2+^-binding domain with conformational properties that are distinctive to each isoform. Our data suggest that the CLR region provides channels with unique functional properties that modulate the rate of passive SR Ca^2+^ leak and confer on RyR1 and RyR3 distinctive [Ca^2+^]_rest_ regulatory properties. The identification of a new Ca^2+^-binding domain of RyRs with a key modulatory role in [Ca^2+^]_rest_ regulation provides new insights into Ca^2+^-mediated regulation of RyRs.

## Abstract 2

This paper reports the finding of a new class of Ca^2+^-binding domain of intracellular Ca^2+^ channels from muscle cells. This domain provides channels with distinctive properties that result in channel-specific modulation of the intracellular resting Ca^2+^ concentration.

## INTRODUCTION

[Ca^2+^]_rest_ (intracellular resting myoplasmic free Ca^2+^ concentration) is delicately regulated by the concerted action of multiple Ca^2+^ pathways that co-ordinate Ca^2+^ fluxes from both sarcolemmal membrane and intracellular Ca^2+^ stores [1–3]. In skeletal muscle, Ca^2+^ release from intracellular Ca^2+^ stores is primarily regulated by RyR1 (type 1 ryanodine receptor), which is under the modulation of multiple endogenous protein regulators [4–6]. A role for RyRs on regulating [Ca^2+^]_rest_ in skeletal muscle was first supported by our studies in dyspedic 1B5 myotubes showing that expression of either RyR1 or RyR3 resulted in significant increase in myoplasmic [Ca^2+^]_rest_ [7]. More recently, Eltit et al. [3] have confirmed these findings, showing that expression of RyR1 in dyspedic primary myotubes accounted for more than half of the total [Ca^2+^]_rest_ measured in wild-type cells. Furthermore, the elevation of [Ca^2+^]_rest_ by expression of RyR1 appears to be the combined effect of both a passive Ca^2+^ leak from a ryanodine-insensitive pool of RyR1 channels (leak channels) and an enhanced basal sarcolemmal Ca^2+^ influx driven by the SR (sarcoplasmic reticulum) Ca^2+^ leak [3,8].

Studies in dysgenic myotubes that lack expression of the DHPR (dihydropyridine receptor) α_1S_ subunit suggest further that negative regulation of RyR1 function by the DHPR also contributes to modulating the passive Ca^2+^ leak responsible for [Ca^2+^]_rest_ [2,9]. In apparent agreement with these findings, the expression of RyR3, which does not interact with DHPR and is therefore not subject to its negative regulation, results in dyspedic myotubes with [Ca^2+^]_rest_ that are significantly higher than those restored by the expression of RyR1 [7]. However, expression of RyR2 in dyspedic myotubes confers [Ca^2+^]_rest_ similar to that of RyR1 [10] despite the fact that, like RyR3, RyR2 also lacks negative regulation by DHPR. These results led us to hypothesize that, in addition to ryanodine-insensitive RyRs and negative regulation by DHPR, other channel properties, which are likely to be unique to each isoform of RyR, also contribute to modulating the passive SR Ca^2+^ leak responsible for [Ca^2+^]_rest_ regulation.

To test this hypothesis, we took advantage of the remarkable differences in channel function and [Ca^2+^]_rest_ modulation conferred by RyR1 and RyR3 [7,11–13] to identify the specific molecular determinants within the primary sequence of RyR1 and RyR3 that define the distinctive [Ca^2+^]_rest_ regulatory properties of each isoform. Using a library of chimaeric RyR3–RyR1 and RyR1–RyR3 receptors expressed in dyspedic myotubes, we show that the ability of the RyR1 and RyR3 to confer isoform-specific [Ca^2+^]_rest_ regulation can be traced to a small domain encompassing amino acids 3770–4007 of the C-terminal region of RyR1 (amino acids 3620–3859 of RyR3), which we referred to as the CLR (Ca^2+^ leak regulatory) region. Functional and structural analyses revealed the CLR domain contains a *bona fide* Ca^2+^-binding domain that modulates the Ca^2+^-sensing properties of RyRs. These data suggest that modulation of [Ca^2+^]_rest_ by RyRs is under the direct control of a novel cation-binding region found within the RyRs with molecular properties unique to each isoform.

## MATERIALS AND METHODS

### Chimaeric RyR3–RyR1 constructs

Chimaeric RyR3–RyR1 and RyR1–RyR3 constructs were designed and cloned as described previously [14–16]. All clones used in the present study have been tested previously and confirmed to express and respond to stimulation by RyR agonists 4-chloro-*m*-cresol and/or caffeine [14–16], thus suggesting that they express functional channels.

### Cell culture, infection and Ca^2+^ imaging

Primary dyspedic myotubes were differentiated in a 96-well plate format as reported previously [15,16]. Myotubes were infected with 2.5×10^4^ HSV-1 (herpes simplex virus 1) virion particles containing wild-type RyR1, wild-type RyR3 or the chimaeric cDNAs constructs [17]. Cells were loaded with 5 μM fura 2/AM (fura 2 acetoxymethyl ester) and imaged at 510 nm with an intensified 10-bit digital CCD (charge-coupled device) camera (XR-Mega-10, Stanford Photonics) using a DG4 multi-wavelength light source as described previously [18]. SR Ca^2+^ content of cultured myotubes was estimated from both peak amplitude and the area under the curve of the Ca^2+^ transient induced by 40 mM caffeine stimulation in the presence of 1 μM thapsigargin. Fluorescent signal was captured from regions of interest within each myotube at 10 frames/s using Piper-control acquisition software (Stanford Photonics) and expressed as the ratio of signal collected at alternating 340 nm/380 nm excitation wavelength. Ca^2+^-entry rates were estimated from the rate of dye quench by Mn^2+^ entry in myotubes loaded with 5 μM fura 2/AM as described previously [3,18].

### [^3^H]Ryanodine binding assay

[^3^H]Ryanodine binding to crude membrane extracts (0.1 mg/ml) was performed at equilibrium (90 min at 37°C) in the presence of 250 mM KCl, 20 mM Hepes (pH 7.4) and 5 nM [^3^H]ryanodine (PerkinElmer Life Sciences) in the presence of a cocktail of protease inhibitors (Complete™, EDTA-free, Roche). Free Ca^2+^ concentrations were obtained by combination of 1 mM EGTA with specific amounts of CaCl_2_ according to calculation with the WEBMAXC Extended program (http://www.stanford.edu/~cpatton/maxc.html). Non-specific binding was determined by incubating the vesicles with 5 μM unlabelled ryanodine. Separation of bound and free ligand was performed by rapid filtration through Whatman GF/B glass fibre filters using a 24-well Brandel cell harvester as described previously [13].

### Resting free Ca^2+^ measurements

[Ca^2+^]_rest_ were obtained by direct measurements on myotubes differentiated in 35-mm-diameter culture dishes using double-barrelled Ca^2+^-selective microelectrodes as described previously [7,18].

### Protein expression and purification

cDNA cassettes encoding either amino acids 3770–4007 of RyR1 (CLR-1) or amino acids 3620–3859 of RyR3 (CLR-3) were cloned into the pCold-II expression vector (Takara™) in-frame downstream of a molecular tag containing a streptavidin-binding peptide and a His_6_ tag. An unrelated protein domain encompassing amino acids 1–233 of RyR1 [R1-(1–233)] containing the same tag was also expressed and used as control protein. Clones were expressed in *Escherichia coli* BL21 strain in combination with pG-KJE8 vector (Takara™) encoding five molecular chaperones to optimize protein folding. Protein expression was induced by incubation with 0.2 mM IPTG for 6–8 h at 16°C in the presence of 1 mg/ml arabinose and 2 ng/ml tetracycline. Cells were then disrupted by sonication in solubilization buffer (20 mM Tris/HCl, pH 7.4, 100 mM KCl, 2 mM EDTA and 0.5% Triton X-100, supplemented with proteinase inhibitors) and soluble proteins were then purified using Strep-trap affinity columns (GE Healthcare) after centrifugation at 100000 ***g*** for 90 min. Proteins were eluted with 3 mM desthiobiotin in 10 mM Tris/HCl (pH 7.4) and then washed/concentrated with 10 mM Tris/HCl (pH 7.4) using filtration units with a 10 kDa molecular-mass cut-off. The purity of the isolated protein domains was evaluated using SDS/PAGE as described previously [4].

### Intrinsic fluorescence spectroscopy and Tb^3+^ fluorescence

Intrinsic fluorescence spectra were recorded at room temperature (24°C) using a QM1 fluorescence spectrophotometer (PTI) with a xenon short arc lamp. Tryptophan fluorescence spectra were collected before and after titration with different concentrations of CaCl_2_ as described previously [19]. Tb^3^-binding affinity of CLR-1 and CLR-3 was obtained by Tb^3+^ FRET analysis as described previously [20,21] (see the Supplementary Online Data at http://www.biochemj.org/bj/460/bj4600261add.htm).

### Circular dichroism

CD spectra were recorded in the far-UV range (190–260 nm) on a Jasco-810 spectropolarimeter at room temperature using a 0.1-cm-pathlength quartz cuvette. The measurements of purified CLR-1 and CLR-3 regions (12–13 μM) were made in 10 mM Tris/HCl (pH 7.4) with either 1 mM EGTA or 0.5 mM CaCl_2_. All spectra documented represent the average of at least 15 scans in which the background signal from the buffer has been subtracted from the sample signals. Thermal denaturation curves were obtained from changes in CD signal at 222 nm between 10°C and 90°C. Measurements were performed in 10 mM Tris/HCl (pH 7.4) with protein concentrations of 10–15 μM. Thermal transition points were calculated by curve fitting as described previously [20].

## RESULTS

### [Ca^2+^]_rest_ level of RyR3–RyR1- and RyR1–RyR3-expressing myotubes

**Figure 1:**
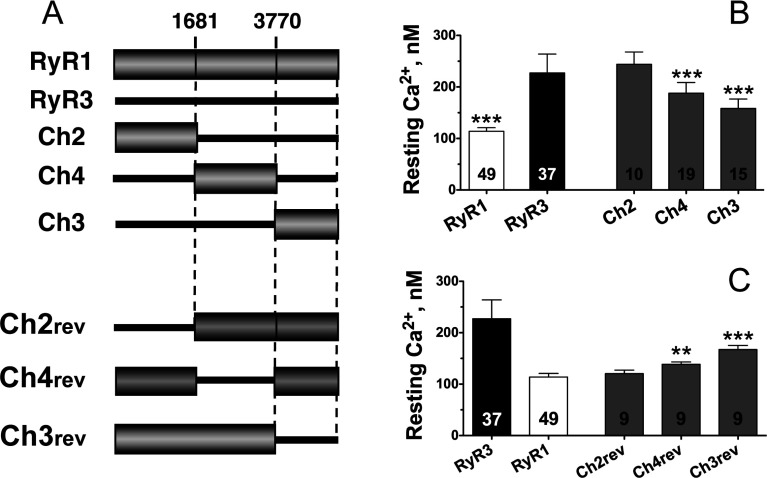
Identification of domains of RyRs important for [Ca^2+^]_rest_ regulation in cultured myotubes (**A**) Schematic representation of RyR3-based and RyR1-based chimaeric receptors expressed in dyspedic myotubes. Numbers indicate amino acid positions in RyR1. (**B**) Average [Ca^2+^]_rest_ values restored by expression of wild-type RyR1, wild-type RyR3 and chimaeric channels Ch2, Ch4 and Ch3. ***P<0.001 compared with wild-type RyR3. (**C**) Average [Ca^2+^]_rest_ values of dyspedic myotubes expressing chimaeras Ch2rev, Ch4rev and Ch3rev representing the exact reverse versions of Ch2, Ch4 and Ch3 respectively. Results are means±S.D. (*n*=2–4). **P<0.01 and ***P<0.001 in comparison with RyR1. Values inside the bars indicate total cells analysed that presented [Ca^2+^] greater than dyspedic myotubes (>50 nM).

To assess whether the differences in myoplasmic [Ca^2+^]_rest_ conferred by RyR1 and RyR3 were associated to specific structural/functional domains of each isoform, we measured [Ca^2+^]_rest_ of dyspedic myotubes expressing a series of chimaeric RyR3–RyR1 constructs spanning the entire primary sequence of RyR1 and RyR3 (Figure 1A). All myotubes presenting [Ca^2+^]_rest_ greater than that of dyspedic myotubes (>50 nM) were considered to be infected and therefore to express the receptor tested [3]. Western blot analysis of infected myotubes indicates that all chimaeric channels tested were expressed at approximately equal levels and showed no differences in expression of the Ca^2+^-handling proteins SERCA1 (sarcoplasmic/endoplasmic reticulum Ca^2+^-ATPase 1) and calsequestrin-1 (Supplementary Figure S1 at http://www.biochemj.org/bj/460/bj4600261add.htm).

Figure 1(B) shows that, whereas the average [Ca^2+^]_rest_ of RyR1-expressing myotubes is approximately 110 nM, RyR3-expressing myotubes had significantly higher average resting free Ca^2+^ levels. Chimaeric constructs Ch4 and Ch3, containing the central and C-terminal region of RyR1 respectively, displayed a significant reduction in [Ca^2+^]_rest_ when compared with wild-type RyR3. Average [Ca^2+^]_rest_ conferred by Ch3-expressing cells was 158±18 nM (*n*=15), a concentration significantly lower than that conferred by chimaera Ch4 [188±20 nM (*n*=19; *P*< 0.001)], but slightly higher than that observed in wild-type RyR1-expressing cells [114±7 nM (*n*=49; *P*<0.001)]. Consistent with these observations, we found that the reverse chimaeric constructs, in which the corresponding Ch4 and Ch3 regions of RyR3 were expressed in a RyR1 background (Ch4rev and Ch3rev), resulted in a significant increase in [Ca^2+^]_rest_ when expressed in dyspedic myotubes (Figures 1A and 1C). Similar to Ch3, the Ch3rev construct had a more dramatic effect on Ca^2+^ homoeostasis and induced higher [Ca^2+^]_rest_ than the Ch4rev region (168±8 nM compared with 138±5 nM for Ch3rev and Ch4rev respectively); however, the [Ca^2+^]_rest_ reached by Ch3rev was lower than that observed in wild-type RyR3-expressing cells (*P*<0.05).

**Figure 2:**
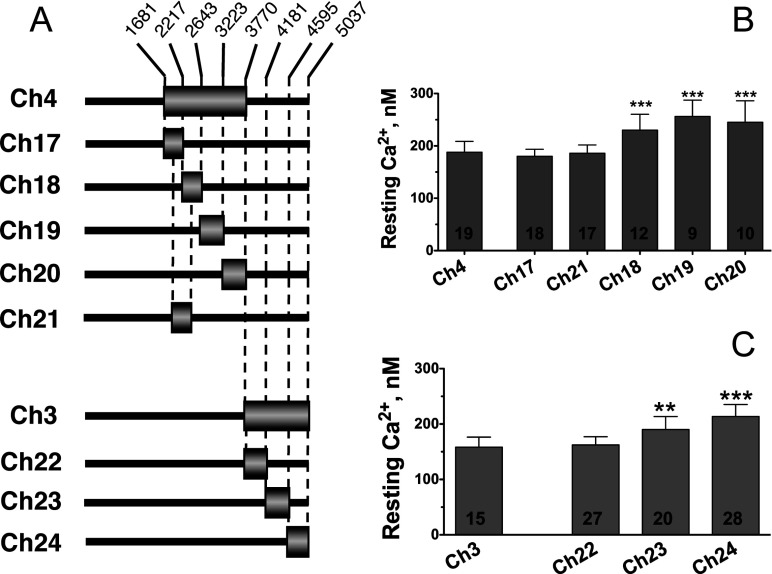
Further localization of the RyR regions responsible for [Ca^2+^]_rest_ regulation (**A**) Schematic representation of chimaeric RyR3–RyR1 receptors containing several contiguous subdomains of region Ch4 and Ch3 of RyR1 into the RyR3 background. Numbers indicate amino acid positions in RyR1. (**B** and **C**) Average [Ca^2+^]_rest_ of myotubes expressing the chimaeric constructs depicted in (**A**). ***P<0.001 compared with Ch4 (**B**). **P<0.01 and ***P<0.001 in comparison with Ch3 (**C**). Values inside the bars indicate total cells analysed. Results are means±S.D. (*n*=2–5).

**Figure 3:**
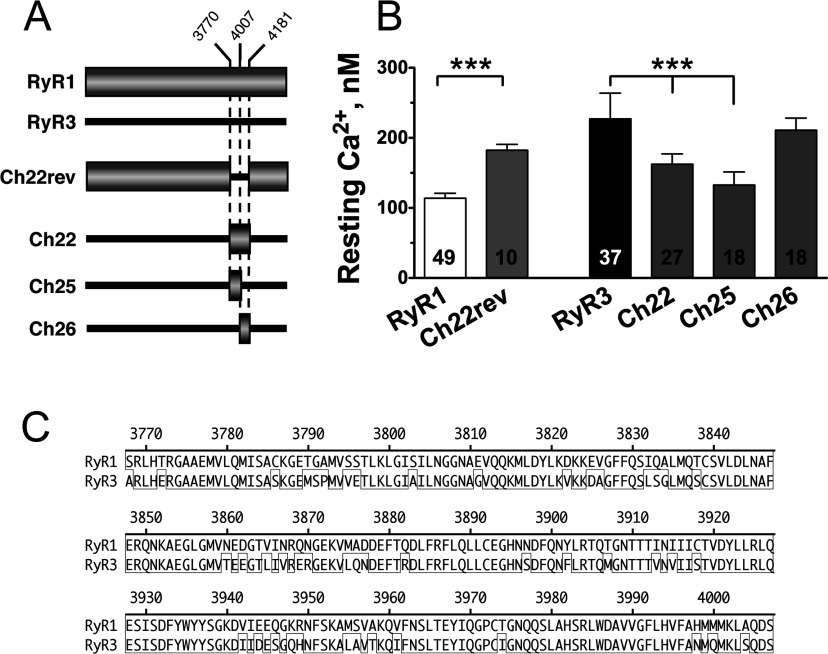
Region Ch22 of RyR3 enhances [Ca^2+^]_rest_ regulation by RyR1 (**A**) Diagram of reverse chimaera Ch22rev-expressing region Ch22 of RyR3 in a RyR1 background. Additional RyR3-based chimaeras further mapping the CLR region of RyR1 are indicated. (**B**) Average [Ca^2+^]_rest_ values of dyspedic myotubes expressing constructs depicted in (**A**). Results are means±S.D. (*n*=2–4). Note that region Ch22 of RyR3 confers on chimaera Ch22rev significantly higher [Ca^2+^]_rest_ regulatory properties than that of the wild-type RyR1 channel, which is similar to that of wild-type RyR3. Further subdivision of region Ch22 into smaller domains Ch25 and Ch26 localized the [Ca^2+^]_rest_ regulatory region at amino acids 3770–4007 of RyR1 and amino acids 3620–3859 of RyR3 (Ch25). (**C**) Amino acid alignment of the CLR region from RyR1 and RyR3 using the Clustal method. Boxed sequence indicate identical residues, and the rule indicates the amino acid position in RyR1.

The critical structural determinant of chimaera Ch4 was mapped further to the smaller overlapping region between chimaeras Ch17 and Ch21 (Figures 2A and 2B), a domain of RyR1 previously found to play a key role in the cross-talk between RyR1 and DHPR [15,16,22]. Subdivision of chimaera Ch3 into three smaller chimaeric constructs showed that the determinant of RyR1 responsible for reduced [Ca^2+^]_rest_ regulation was confined to a smaller domain of the C-terminal region, within amino acids 3770–4180 of RyR1 (chimaera Ch22, Figures 2A and 2C). To demonstrate that the reduction in [Ca^2+^]_rest_ regulation by Ch22 was not due to a conformational change, we also tested the reverse chimaera Ch22rev, in which the region Ch22 of RyR3 was expressed into the corresponding region of RyR1 (Figure 3A). Figure 3(B) shows that, as expected, expression of Ch22rev in dyspedic myotubes resulted in [Ca^2+^]_rest_ that were significantly higher than those observed for wild-type RyR1-expressing myotubes and much closer to those restored by wild-type RyR3 [182±8 nM (*n*=10) for Ch22rev compared with 114±7 nM (*n*=49) for RyR1 (*P*<0.001)]. The average [Ca^2+^]_rest_ restored by Ch22rev, however, was still lower than those restored by wild-type RyR3 (*P*<0.01).

Further analysis narrowed down the location of the [Ca^2+^]_rest_ regulatory domain of region Ch22 of RyR1 to its N-terminal half (Ch25 in Figures 3A and 3C). Dyspedic myotubes expressing Ch25 showed an average [Ca^2+^]_rest_ value of 133±18 nM (*n*=18) that was significantly lower than that observed with the larger Ch22 region [162±14 nM (*n*=27; *P*<0.01)]. [Ca^2+^]_rest_ displayed by Ch25-expressing cells was not statistically different from those observed in wild-type RyR1-expressing myotubes (*P* > 0.05; Figure 3B). Overall, these data show that region Ch25, encompassing amino acids 3770–4007 of RyR1 (amino acids 3620–3859 of RyR3, Figure 3C), holds a structural determinant of RyR sequence that confers the Ca^2+^ channel with resting Ca^2+^-regulatory properties, which are specific to each isoform. Because of the role of this domain in modulating the rate of Ca^2+^ leak through the channel (see below), for simplicity we refer to this domain of RyRs as the CLR region.

### Effect of the CLR region on RyRs Ca^2+^ channel function

**Figure 4:**
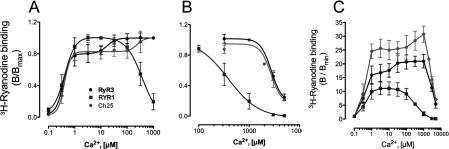
CLR region modulates the Ca^2+^-dependence function of RyRs RyR channel function was assessed by specific [^3^H]ryanodine binding to crude membrane preparation from HEK-293 cells permanently transformed with wild-type RyR1, wild-type RyR3 or chimaera Ch25. (**A**) Normalized Ca^2+^-activation curve for RyR1 (squares), RyR3 (dark circles) and Ch25 (grey circles). RyR1 is fitted to a monophasic Hill equation, whereas RyR3 and Ch25 are fitted to the biphasic Hill equations. (**B**) Normalized Ca^2+^-inhibition curves for wild-type RyR1, wild-type RyR3 and Ch25 fitted to a monophasic equation. (**C**) Ca^2+^-dependence of [^3^H]ryanodine binding expressed as the fraction of *B*_min_ showing that channel activity of chimaera Ch25 preserves the same functional profile of RyR3. Activation and inhibition profiles are the combined result of four to six experiments performed in duplicate or triplicate from multiple HEK-293 membrane preparations. Results are means±S.E.M. (*n*=4–6).

Several groups have reported significant differences in Ca^2+^ channel properties between RyR1 and RyR3 [11–13,23]. To assess whether the effect of the CLR region of RyRs on [Ca^2+^]_rest_ regulation of dyspedic myotubes was associated to alterations in channel properties, we further analysed the Ca^2+^-dependence of the chimaeric construct Ch25 using [^3^H]ryanodine-binding studies. Figure 4(A) shows the average Ca^2+^-dependence of [^3^H]ryanodine binding to membrane fractions from HEK (human embryonic kidney)-293 cells expressing either wild-type channels or chimaeric construct Ch25. As reported previously, Ca^2+^-dependence profiles of wild-type RyR1 and wild-type RyR3 indicate monophasic activation for wild-type RyR1 [EC_50_=0.41±0.18 μM (*n*=6)] and a biphasic activation profile for wild-type RyR3 [EC_50(1)_=0.39±0.04 μM and EC_50(2)_=35.1±18 μM (*n*=6)] with a plateau between 1 and 10 μM. Consistent with its predominant RyR3 background, the Ca^2+^-activation curve of chimaera Ch25 showed a biphasic activation profile, but with a more exacerbated biphasic Ca^2+^ response and a wider activation plateau (1–100 μM; Figure 4) than wild-type RyR3 [EC_50(1)_=0.36±0.03 μM and EC_50(2)_=465.7±120 μM (*n*=4)]. [^3^H]Ryanodine-binding analysis of Ca^2+^ inhibition revealed that Ca^2+^-sensitivity for inactivation of RyR3 was 6–7-fold lower than that of RyR1 (IC_50_=0.44±0.22 mM for RyR1 compared with IC_50_=2.68±0.77 mM for RyR3). Analysis of Ca^2+^ inactivation for chimaera Ch25 showed a significantly reduced sensitivity to Ca^2+^ inhibition compared with wild-type RyR1, but similar to that of wild-type RyR3 (IC_50_=3.01±0.97 mM, Figure 4B). Comparison of the [^3^H]ryanodine-binding data expressed as a fraction of the basal activity (*B*/*B*_min_) shows important differences in channel function between wild-type RyR1 and wild-type RyR3, with RyR3 reaching noticeably higher activation levels than RyR1 (Figure 4C). Like RyR3, chimaera Ch25 also displays high activation levels, suggesting that, despite changes in its Ca^2+^-activating profile, chimaera Ch25 preserves most of the channel function characteristic of RyR3. The curve fit and statistics of Ca^2+^ activation and inhibition for wild-type and chimaeric receptors are presented in Tables 1 and 2. Overall, these data suggest that substitution of the CLR region of RyRs results in significant changes in the Ca^2+^ activation properties of the chimaeric channels.

**Table 1:** Curve fit statistics for [^3^H]ryanodine-binding analyses of Ca^2+^ activation Results are means±S.D. *P<0.001 (one-way ANOVA with Tukey's post-hoc analysis).

Construct	EC_50(1)_ (μM)	EC_50(2)_ (μM)	*n*
RyR1	0.41±0.18	N/A	6
RyR3	0.39±0.04	35.1±18*	6
Ch25	0.36±0.03	465.7±120*	4

**Table 2:** Curve fit statistics for [^3^H]ryanodine-binding analyses of Ca^2+^ inhibition Results are means±S.D.

Construct	IC_50_ (mM)	*n*
RyR1	0.44±0.22	4
RyR3	2.68±0.77	4
Ch25	3.01±0.97	5

### Effect of the CLR region on myoplasmic Ca^2+^ fluxes

**Figure 5:**
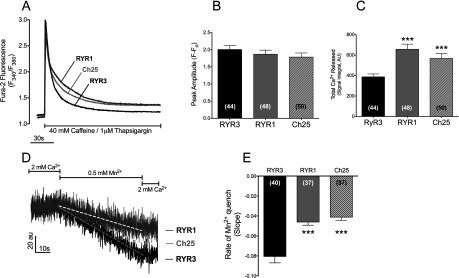
Exchange of CLR regions among isoforms of RyR modulate SR Ca^2+^ leak and sarcolemmal Ca^2+^ entry (**A**) Average caffeine-induced Ca^2+^-release transients of fura 2-loaded dyspedic myotubes expressing wild-type RyR1, wild-type RyR3 or chimaera Ch25. S.D. of the traces has been omitted for clarity. Average peak Ca^2+^ transient amplitude (**B**) and total Ca^2+^ release (**C**) are shown for each population of myotubes. ***P<0.001 in comparison with wild-type RyR3. (**D**) Representative traces of Mn^2+^ quench of fura 2 fluorescence of RyR1- and RyR3-expressing myotubes showing differences in resting sarcolemmal Ca^2+^ entry among isoforms. (**E**) Average rate of Mn^2+^ quench, calculated from the slope of the fluorescent signal (broken lines), is shown for each set of constructs. Results are means±S.E.M. for a total of nine or ten individual cultured wells (*n*=3). Values in parentheses indicate total myotubes analysed.

Because of the importance of Ca^2+^ flux equilibrium to myoplasmic [Ca^2+^]_rest_ regulation, we also assessed whether CLR-mediated changes in the Ca^2+^-sensing properties of RyRs had an effect on the rate of SR Ca^2+^ leak or rate of sarcolemmal Ca^2+^ entry in dyspedic myotubes expressing either wild-type or Ch25 constructs. SR Ca^2+^ leak was estimated using the differences in SR Ca^2+^ load measured by the magnitude of Ca^2+^ release induced by a 40 mM caffeine challenge in the presence of the SERCA1 pump inhibitor thapsigargin. Figure 5(A) shows that myotubes expressing wild-type RyR1 generated caffeine-induced Ca^2+^ release transients that were significantly larger than those of myotubes expressing wild-type RyR3. Analysis of average Ca^2+^ transient revealed no significant differences in peak transient amplitude between isoforms (Figure 5B). However, the total Ca^2+^ released in RyR3-expressing myotubes, which was measured as the integral of the fluorescent signal, was found to be significantly reduced (Figure 5C), indicating an enhanced SR Ca^2+^ leak. Consistent with this smaller SR Ca^2+^ load, RyR3-expressing myotubes also displayed significantly higher rates of Mn^2+^ quench than RyR1-expressing myotubes (Figures 5D and 5E) revealing increased rates of sarcolemmal Ca^2+^ entry at rest. By comparison, Ch25-expressing cells displayed SR Ca^2+^ release at levels between RyR1 and RyR3, showing similar peak Ca^2+^ transient amplitude, but with significantly increased total Ca^2+^ release compared with wild-type RyR3-expressing cells (Figures 5A–5C). Likewise, Ch25-expressing myotubes showed rates of resting Mn^2+^ quench similar to those of RyR1-expressing cells and significantly lower than those of RyR3-expressing myotubes (Figures 5D and 5E). These data suggest that myotubes expressing Ch25 and RyR1 present similar rates of SR Ca^2+^ leak and that the differences in SR Ca^2+^ load observed between Ch25 and RyR1 (Figure 5C) are likely to be the result of reduced sensitivity to Ca^2+^ inhibition displayed by Ch25 (Figure 4) that allows for a faster emptying of the Ca^2+^ stores than RyR1. These findings are consistent with previous studies indicating increased basal channel function of RyR3 [24–27] and suggest that the insertion of the CLR region of RyR1 into the RyR3 sequence (chimaera Ch25) results in less leaky channels.

### Expression and purification of the CLR region

**Figure 6:**
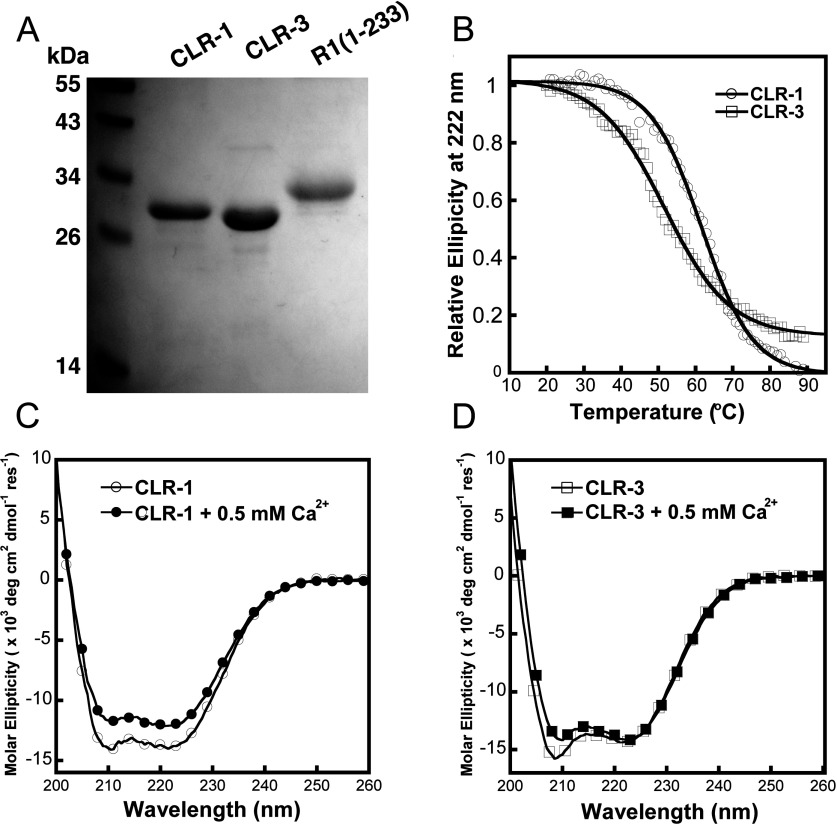
Purification and conformational analysis of the CLR region of RyR1 and RyR3 (**A**) Coomassie Blue-stained SDS/PAGE gel loaded with 2 μg/lane CLR-1, 4 μg/lane CLR-3 and 3 μg/lane R1-(1–233) purified from *E. coli* BL21 cells. (**B**) Representative thermal stability curves for CLR-1 and CLR-3 showing changes in CD signal at 222 nm (*n*=2). (**C** and **D**) Comparison of far-UV CD spectra of purified CLR-1 (circles) and CLR-3 (squares) regions in the presence of 1 mM EGTA (unfilled symbols) indicating high α-helix content and small differences in secondary/tertiary structure between isoforms. Note the small but reproducible decrease in ellipticity of the CD spectra in the presence of 0.5 mM Ca^2+^ (filled symbols).

[^3^H]Ryanodine-binding studies suggest the CLR region may be involved in Ca^2+^-mediated activation of RyRs. To evaluate a direct role of this region in modulating the Ca^2+^-sensing properties of RyRs, a fragment comprising the CLR region of RyR1 (amino acids 3770–4007, herein CLR-1) and the corresponding fragment in RyR3 (amino acids 3620–3859, herein CLR-3), were expressed in *E. coli* BL21 cells and analysed for their ability to bind Ca^2+^. In order to minimize misfolding of the protein domains, the CLR fragments were expressed in the presence of five chaperone proteins, to assist in protein folding, and then purified from the soluble fraction of the cell homogenate to avoid using inclusion bodies. All purified domains, including control region R1-(1–233), showed approximately 95% purity and molecular masses of approximately 30 kDa, consistent with their predicted 25–27 kDa molecular mass (Figure 6A).

### Conformational analysis of CLR domains

Far-UV CD analysis of CLR-1 and CLR-3 indicate that both purified protein domains exhibit helical conformation (Figures 6C and 6D). The well-folded secondary structure of these fragments is consistent with the fact that intrinsic tryptophan fluorescence emission of the CLR regions peak at 330 nm (see Figure 8), and suggests that the tryptophan residues are largely buried within this folded conformation [28]. Moreover, analysis of thermal transition revealed important differences in thermal stability between CLR-1 and CLR-3 (Figure 6B). CLR-1 appeared to be more stable than CLR-3 with approximately 10°C difference in melting temperature (*T*_m_=61.5±0.2°C and 51.7±0.3°C for CLR-1 and CLR-3 respectively) and a more co-operative thermal unfolding curve than RyR3. These data are consistent with isoform-specific differences in domain packing and suggest that the CLR region of RyR3 displays a higher degree of molecular flexibility than its RyR1 counterpart.

### Metal-binding properties of the CLR domain

**Figure 7:**
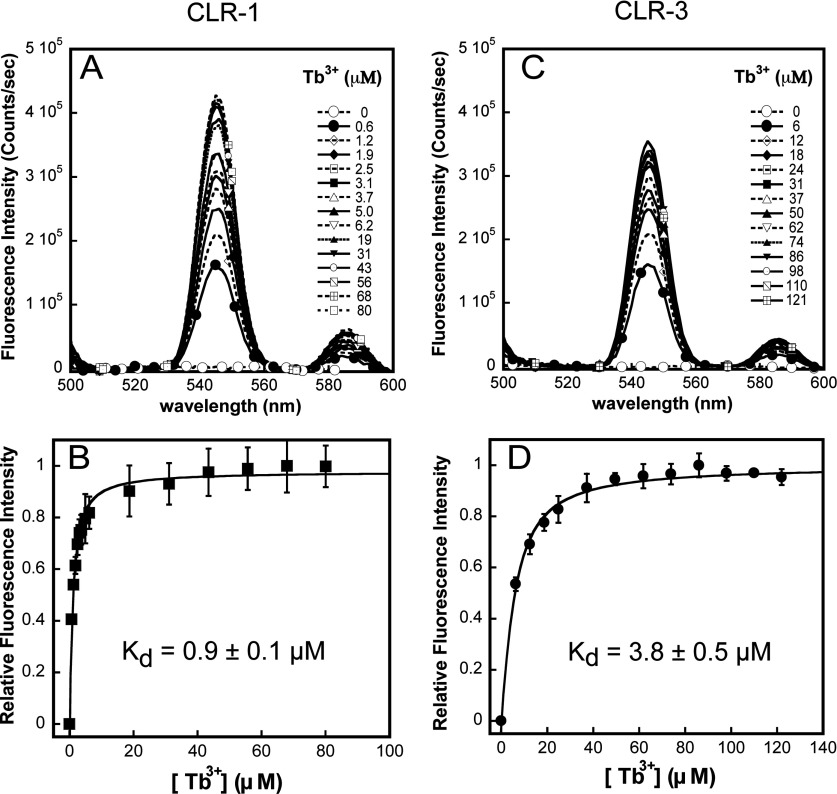
CLR-1 and CLR-3 domains bind Tb^3+^ with high affinity Representative Tb^3+^ fluorescence spectra of purified CLR-1 (**A**) and CLR-3 (**C**) regions showing an increase in fluorescence intensity at 545 nm upon titration with Tb^3+^ (excitation at 280 nm). Tb^3+^ binding to CLR-1 (**B**) and CLR-3 (**D**) domains is expressed as the average change in relative fluorescence intensity ([*F*_i_−*F*_min_]/[*F*_max_−*F*_min_]). Data were fitted to monophasic binding curves using eqn (S3) (see the Supplementary Online Data at http://www.biochemj.org/bj/460/bj4600261add.htm). Results are means±S.D. (*n*=3).

Upon addition of 0.5 mM Ca^2+^, both domain fragments displayed a small, but reproducible, decrease in mean residue ellipticity (205–225 nm) indicating Ca^2+^ changes in secondary structure (Figures 6C and 6D), therefore Ca^2+^ interaction with the CLR domains. Interestingly, the effects of Ca^2+^ in secondary structure of CLR-1 were significantly more pronounced than the changes observed in CLR-3. Direct cation binding was assessed using Tb^3+^ fluorescence analysis. This binding assay is commonly used to determine Ca^2+^-binding sites because Tb^3+^ has a similar ionic radius and metal–co-ordination chemistry to that of Ca^2+^, while allowing for quantitative analysis [29,30]. As shown in Figure 7, titration of the purified CLR-1 and CLR-3 domains with micromolar concentrations of TbCl_3_ resulted in a significant increase in fluorescent signal at 545 nm when excited at 280 nm. Both CLR domains showed monophasic binding curves with CLR-1 displaying significantly higher binding affinity for Tb^3+^ than domain CLR-3 [*K*_d_=0.86±0.07 μM for CLR-1 compared with 3.8±0.5 μM for CLR-3 (*n*=3; *P*<0.001); Figures 7B and 7D and Table 2], supporting further the existence of a cation-binding pocket with moderate affinity within the primary structure of the CLR region of RyR1 and RyR3.

**Figure 8:**
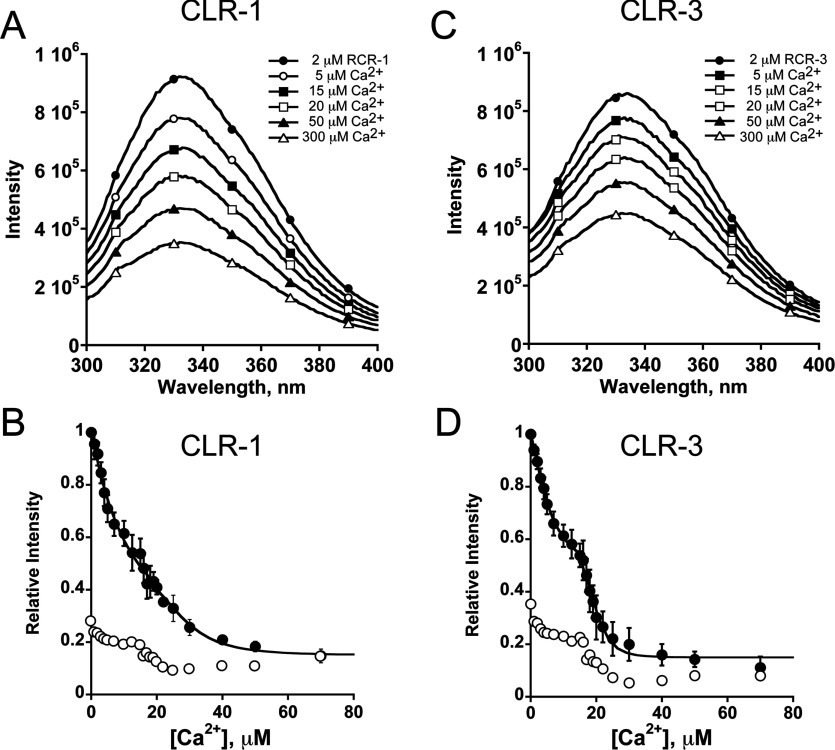
Characterization of the Ca^2+^-binding properties of CLR-1 and CLR-3 Representative fluorescence emission spectra showing a Ca^2+^-induced decrease in intrinsic tryptophan fluorescence intensity of CLR-1 (**A**) and CLR-3 (**C**) domains excited at 280 nm (filled circles). Ca^2+^ titration curves were fitted to a biphasic binding process using eqns (S1) and (S2) (see the Supplementary Online Data at http://www.biochemj.org/bj/460/bj4600261add.htm) (**B** and **D**). Results are mean±S.D. relative fluorescence intensity (*n*=3). Ca^2+^-induced changes in relative fluorescence intensity of control protein R1-(1–233) (**B** and **D**, unfilled circles) are shown as fractions of the maximal response of either CLR-1 or CLR-3.

To characterize further the Ca^2+^-binding properties of the cation-binding pocket, we next analysed Ca^2+^-induced conformational changes in the purified domains by monitoring intrinsic tryptophan fluorescence. As shown in Figures 8(A) and 8(C), upon titrating with increasing concentrations of Ca^2+^ both domain fragments displayed a large decrease in tryptophan fluorescence. By comparison, Ca^2+^ titration of a control unrelated fragment of RyR1 [R1-(1–233)], containing amino acids 1–233 of RyR1, showed only marginal fluorescence changes upon Ca^2+^ addition (Figures 8B and 8D). This region of RyR1 has been crystallized previously and its atomic structure has confirmed the absence of formal cation-binding sites [31]. These fluorescence data are consistent with the Ca^2+^-induced conformational changes revealed by the CD spectroscopy assay and suggest that Ca^2+^ binding detected by the tryptophan fluorescence is specific to the CLR domains.

Unlike the Tb^3+^ titration assay, Ca^2+^ titration of tryptophan fluorescence was better fitted to a biphasic Ca^2+^-binding process (Figures 8B and 8D). This biphasic profile was particularly evident in CLR-3 with Ca^2+^-binding affinities in the micromolar range (*K*_d1_=7.3±0.3 μM and *K*_d2_=18.4±1.6 μM). The biphasic binding profile of domain CLR-1 was less pronounced with *K*_d1_ of 11.0±1.8 μM and *K*_d2_ of 21.6±2.7 μM. Whereas average *K*_d1_ showed a slight, but significant, difference between CLR-1 and CLR-3, there was no difference seen in average *K*_d2_, suggesting that the CLR region of RyR1 and RyR3 may present slightly different Ca^2+^-binding properties (Table 3). The high Hill coefficients of the CLR domains are suggestive of several ligand-binding sites; however, other factors such as the ligand-induced change in protein conformation may also contribute to this high level of co-operativity [32].

**Table 3:** Curve fit statistics for Tb^3+^- and Ca^2+^-binding analyses of purified CLR domains Results are means±S.D. *P<0.05 compared with *K*_d2_; ***P<0.001 compared with CLR-1 (Student's *t* test).

		Ca^2+^ (μM)		
Domain	Tb^3+^ (μM) *K*_d_	*K*_d1_	*K*_d2_	Hill coefficient
CLR-1	0.9±0.1	11.0±1.8*	21.6±2.7	4.9±0.5
CLR-3	3.8±0.5***	7.3±0.3*	18.4±1.6	6.7±1.8

## DISCUSSION

In a previous study, we showed that expression of RyR1 and RyR3 in cultured dyspedic myotubes has a dramatic effect in the resting Ca^2+^ regulation where RyR3 restored myoplasmic [Ca^2+^]_rest_ to significantly higher Ca^2+^ levels than RyR1 [7]. In the present study, we used a series of chimaeric RyR channels spanning the entire sequence of RyR1 and RyR3 in order to localize and characterize the molecular determinant of each isoform responsible for their differential effect on [Ca^2+^]_rest_.

### Location of the resting Ca^2+^-regulatory domain of RyRs

We found that the ability of RyR1 and RyR3 to differentially modulate [Ca^2+^]_rest_ can be traced to two regions of the primary sequence: one is the overlapping region between chimaeras Ch17 and Ch21 (17/21, amino acids 1924–2217 of RyR1 and 1798–2082 of RyR3) and the other the CLR region encompassing amino acids 3770–4007 of RyR1 (amino acids 3620–3859 of RyR3). The impact of region 17/21 on [Ca^2+^]_rest_ regulation was shown to be less dramatic than that of the CLR region in both the RyR3-based (Ch4) and the RyR1-based (Ch4rev) chimaeric constructs. Our previous studies of these chimaeric receptors have shown that the RyR1 region encompassing region 17/21 is directly involved in the cross-talk between DHPR and RyR1, and confers RyR3 with the ability to restore DHPR tetrad formation, DHPR retrograde signalling and excitation–contraction coupling [15,16,22]. Therefore it is likely that the mild reduction in [Ca^2+^]_rest_ observed in dyspedic myotubes expressing Ch4, Ch21 or Ch17 could be related to an enhanced orthograde regulation by DHPR that inhibited RyR1 function, thereby restricting overall SR Ca^2+^-leak, similar to the mechanism described by Eltit et al. [2,3]. Nonetheless, an effect of altered Ca^2+^ regulation by Ch4, Ch21 and Ch17 on the rates of SR Ca^2+^ leak and its subsequent effect on [Ca^2+^]_rest_ should not be ruled out as all these chimaeric constructs have shown to affect both Ca^2+^-activation and Ca^2+^-inhibition profiles of the channel [13].

The CLR regions of RyR1 and RyR3 were shown to have a more dramatic effect on Ca^2+^ homoeostasis conferring on chimaeric receptors [Ca^2+^]_rest_-regulatory properties distinctive of the specific CLR sequence being expressed, regardless of the overall amino acid background. In contrast with region 17/21, it is unlikely that the effect the CLR region has on [Ca^2+^]_rest_ could be related to orthograde regulation by DHPR, as cumulative evidence indicates that the C-terminal region of RyR1 is not involved in cross-talk with the DHPR complex. Our previous studies involving several chimaeric RyR3–RyR1 channels, including Ch25 used in the present study, have shown that exchange of various RyR domains containing the CLR region had a negligible effect on either excitation–contraction coupling [14] or DHPR Ca^2+^ current densities [15,16,22], suggesting that the RyR region encompassing the CLR region is not involved directly in the DHPR–RyR interaction.

Importantly, and consistent with our previous studies in wild-type RyR3 and wild-type RyR1 [7], Western blot analysis indicates that all chimaeric constructs displayed similar expression levels when transfected in dyspedic myotubes. Therefore changes in [Ca^2+^]_rest_ like the one caused by exchange of the CLR region could not be explained simply by changes in expression levels of the chimaeric channels.

Chronically elevated [Ca^2+^]_rest_ is a common feature of skeletal muscle cells expressing RyR1 mutations linked to MHS (malignant hyperthermia syndrome). Extensive functional analysis of muscle cells expressing numerous MHS-linked mutations of RyR1 has revealed that elevated [Ca^2+^]_rest_ is often associated with alteration of Ca^2+^ and Mg^2+^ regulation of RyR1 [3,8,33–38], with a number of these mutations being confirmed to result in increased basal Ca^2+^ channel function that leads to enhanced passive SR Ca^2+^ leak [8,34,36,39]. The reverse mechanism seems to operate in chimaera Ch25 where the insertion of the more stable CLR region of RyR1 into the RyR3 sequence appeared to attenuate the enhanced basal channel function of RyR3, causing myotubes to decrease both overall SR Ca^2+^ leak and sarcolemmal Ca^2+^ entry, resulting in a subsequent reduction of [Ca^2+^]_rest_. These results are also in agreement with the cell boundary theorem that predicts permanent changes in cytosolic Ca^2+^ concentration as a result of steady changes of sarcolemmal Ca^2+^ fluxes [1].

### Molecular properties of the CLR region

The identification of *bona fide* Ca^2+^-binding sites in the CLR region of RyR1 and RyR3 with Ca^2+^ affinities within the physiological range seems in agreement with a role for the CLR region in Ca^2+^-mediated regulation of RyRs. However, whether Ca^2+^ binding to the CLR region is involved directly in Ca^2+^-mediated activation of RyRs needs further study. Our *in vitro* biochemical characterization (Ca^2+^ titration) of the isolated CLR domains suggests that Ca^2+^-binding affinities of CLR-1 and CLR-3 are similar. It therefore seems unlikely that the shift of Ca^2+^-sensing properties resulting from the exchange of CLR region in chimaera Ch25 could be explain by the overall Ca^2+^-binding properties of the CLR region alone. Instead, our conformational analysis suggests significant differences in overall molecular conformation between the CLR regions of RyR1 and RyR3. Differences in the secondary/tertiary structure between CLR-1 and CLR-3 are supported by CD analysis, which revealed slight differences in helical content among domains (Figures 6C and 6D). This difference in folded conformation is also evident from the differential effect of Ca^2+^ in the CD spectra. Indeed, whereas Ca^2+^ addition resulted in changes in the overall conformation of CLR-1, it had significantly less effect on the folded structure of CLR-3. This is supported further by thermal transition studies (Figure 6B) that revealed important differences in thermal stability and unfolding co-operativity between CLR domains, with the overall structure of CLR-3 being less stable and packed than that of CLR-1.

An intriguing result was the differences in binding profile (monophasic compared with biphasic) and affinity of the cation-binding pocket detected by Tb^3+^- and Ca^2+^-binding assays. These differences probably arise from the intrinsic differences in which each assay reads out cation–protein interaction. Whereas changes in tryptophan fluorescence are the result of global conformational changes of the CLR domain, the Tb^3+^ fluorescence signal is the result of energy transfer between nearby tryptophan residues and Tb^3+^, and therefore primarily reflects local events that are highly sensitive to molecular distance. A monophasic binding profile like the one observed during Tb^3+^ titration could stem from the fact that tryptophan residues were present in close proximity to only one of the Tb^3+^-binding sites. This seems consistent with the actual location of the two tryptophan residues of the CLR region, which are found close to each other and clustered towards the C-terminal end of the domain (Figure 3C).

At the functional level, biphasic Ca^2+^-activating profiles like the one observed in our [^3^H]ryanodine-binding studies have been reported previously in RyR3 [13] and RyR2 [40,41] and are consistent with our hypothesis that activation of RyRs may involve at least two moderately co-operative Ca^2+^-activation sites [13]. The binding of Ca^2+^ to multiple sites in a highly co-operative manner have been shown to facilitate protein responses to small changes in Ca^2+^ concentration [42,43]. The high degree of co-operativity to Ca^2+^-binding showed by the CLR domains (Table 3) and the effects of these domains in the Ca^2+^-activation profile of chimaeric receptors are consistent with this idea. These data suggest that Ca^2+^-dependent activation of RyR channels may be a complex event possibly involving multiple Ca^2+^-binding sites.

It is worth noting that the RyR1 sequence around the CLR has been previously associated with Ca^2+^ regulation with several potential Ca^2+^-binding and/or Ca^2+^-regulatory sites being reported in a number of studies (Supplementary Figure S2 at http://www.biochemj.org/bj/460/bj4600261add.htm). Even though the nature of the Ca^2+^-binding sites at the CLR region is currently unknown, it appears that the amino acid ligands that conform the Ca^2+^-binding domain(s), do not group as continuous canonical EF-hand motif, but rather as a discontinuous array. This is not surprising as an analysis of the atomic structure of more than 1600 Ca^2+^-binding domains have shown that discontinuous non-EF-hand binding sites account for more than 90% of the known structures [44]. To our knowledge, this is the first report linking amino acids 3770–4007 of RyR1 (3620–3859 of RyR3) to either direct Ca^2+^-binding or [Ca^2+^]_rest_ regulation. Previous studies have associated this same region with interaction to S100A1 [45], a Ca^2+^-binding protein known to modulate Ca^2+^ cycling in skeletal muscle through its interaction with the calmodulin-binding site of RyR1 [46,47]. However, it is unlikely that the effects of the CLR region in [Ca^2+^]_rest_ regulation observed in our study could be linked to binding of S100A1 because S100A1-null muscle fibres appear to present normal [Ca^2+^]_rest_ regulation [46].

Overall, our data revealed that the region of amino acids 3770–4007 of RyR1 (amino acids 3620–3859 of RyR3) encompasses a novel Ca^2+^-binding domain that provide RyR with unique conformational properties that define their distinctive modulatory role in [Ca^2+^]_rest_ regulation. Because this modulatory role appears to be linked directly to the ability of RyRs to regulate passive SR Ca^2+^ leak [2,3], it is conceivable that the effect of the CLR region on [Ca^2+^]_rest_ could result from changes in RyR susceptibility to adopt the leak conformation. Our data, however, do not rule out the possibility that changes in gating properties of the functional pool of RyRs could also contribute to modulate the overall SR Ca^2+^ content that drives sarcolemmal Ca^2+^ fluxes and thereby [Ca^2+^]_rest_ regulation. Lastly, the identification of this new Ca^2+^-binding domain in RyRs, which was found to play a key role in modulating myoplasmic [Ca^2+^]_rest,_ provides new insights into Ca^2+^-mediated regulation of RyRs function.

## Online data

Supplementary data
