# Supplementary material for: Myoplasmic resting Ca2+ regulation by ryanodine receptors is under the control of a novel Ca2+-binding region of the receptor
Source: Biochem J. 2014 May 13;460(Pt 2):261–71. doi: 10.1042/BJ20131553 (PMC4019983; doi:10.1042/BJ20131553)
Supplement: Supplementary data [file bj4600261add.pdf]

Biochem. J. (2014) 460, 261–271 (Printed in Great Britain) doi:10.1042/BJ20131553

SUPPLEMENTARY ONLINE DATA

Myoplasmic resting Ca^{2+} regulation by ryanodine receptors is under the control of a novel Ca^{2+}-binding region of the receptor

Yanyi CHEN*[1], Shenghui XUE*[1], Juan ZOU*, Jose R. LOPEZ†, Jenny J. YANG*[2] and Claudio F. PEREZ‡[2]

*Department of Chemistry, Center for Diagnostics and Therapeutics, Georgia State University, 50 Decatur Street, NSC 552, Atlanta, GA 30303, U.S.A.
†Department of Molecular Biosciences, School of Veterinary Medicine, University of California, Davis, 1089 Veterinary Medicine Drive, Davis, CA 95616, U.S.A.
‡Department of Anesthesiology, Perioperative and Pain Medicine, Brigham and Women's Hospital, Harvard Medical School, 20 Shattuck Street, Boston, MA 02115, U.S.A.

www.biochemj.org Biochemical Journal

SUPPLEMENTARY METHODS

Tryptophan and Tb^{3+} fluorescence spectroscopy

Ca^{2+}-induced fluorescence changes of CLR-1 or CLR-3 (2 μM) were monitored in the presence of 10 mM Pipes (pH 6.8) and 100 mM KCl. All solutions were dialysed against Chelex-100 for at least 24 h to remove contaminant cations. Intrinsic tryptophan fluorescence was monitored using excitation at 280 nm and emission between 300 and 400 nm with 2–4-nm band passes. Ca^{2+} dissociation constants of the CLR domains were calculated separately for each binding phase. The first binding phase was fitted to eqn (S1):

$$f = \frac{([P]_T + [M]_T + K_{d1}) - \sqrt{([P]_T + [M]_T + K_{d1})^2 - 4[P]_T[M]_T}}{2[P]_T} \quad \text{(S1)}$$

where f is the fractional change, K_{d1} is the dissociation constant, and $[P]_T$ and $[M]_T$ are the total concentration of protein and Ca^{2+} respectively. The second co-operative Ca^{2+}-binding phase was fitted to eqn (S2):

$$\Delta S = \Delta S_1 + \Delta S_2 \frac{[M]^h}{K_{d2}^h + [M]^h} \quad \text{(S2)}$$

where ΔS is the total fluorescence signal change, ΔS_1 and ΔS_2 are the signal changes in the first and second binding phases respectively, [M] is the Ca^{2+} concentration, h is the Hill coefficient, and K_{d2} is the Ca^{2+} dissociation constant for the second binding phase.

Tb^{3+}-binding affinity of CLR-1 and CLR-3 was obtained by Tb^{3+} FRET analysis as described previously [1,2]. Briefly, 2 μM purified CLR-1 or CLR-3 was resuspended in binding solution (10 mM Pipes, pH 6.8, and 100 mM KCl) and titrated with increasing concentrations of $TbCl_3$. FRET was then acquired by excitation of tryptophan (donor) at 280 nm and collection of Tb^{3+} fluorescence emission (acceptor) between 500 and 600 nm using a glass filter to cut off emission below 400 nm. Specific changes in Tb^{3+} fluorescent signal were obtained from the emission intensity at 545 nm after removal of free Tb^{3+} background signal. The Tb^{3+} dissociation constant for CLR-1 or CLR-3 was obtained by fitting the normalized fluorescent intensity data to eqn (S3):

$$f = \frac{([P]_T + [M]_T + K_d) - \sqrt{([P]_T + [M]_T + K_d)^2 - 4[P]_T[M]_T}}{2[P]_T} \quad \text{(S3)}$$

where f is the fractional change, K_d is the dissociation constant, and $[P]_T$ and $[M]_T$ are the total concentration of protein and Tb^{3+} respectively.

Cell membrane isolation and immunoblotting

Crude membrane preparations were made from three 10-cm-diameter plates of 5-day differentiated dyspedic myotubes 36 h after infection with (1.5–2) $\times 10^6$ virion particles. Myotubes were harvested in PBS and centrifuged at 250 ***g*** for 10 min. Cell pellets were resuspended in buffer consisting of 250 mM sucrose and 10 mM Hepes (pH 7.4), supplemented with 1 mM EDTA, 10 μg/ml leupeptin, 0.7 μg/ml pepstatin A, 5 μg/ml aprotinin and 0.1 mM Pefabloc SC and then homogenized using a Tissue-Tearor™ cell disrupter (Biospect Products). Whole-cell homogenates were centrifuged at 1500 ***g*** for 20 min and the supernatants then collected and recentrifuged at 100 000 ***g*** for 60 min at 4 °C. Membrane pellets were finally resuspended in 250 mM sucrose and 20 mM Hepes (pH 7.4), quick frozen in liquid N_2 and stored at -80 °C. Proteins (25–30 μg/lane) were separated in discontinuous SDS/PAGE 7–12 % or 7–15 % gels [3,4] and then electroblotted on to PVDF membranes for 90 min at 50 V. Membrane sections containing the protein to be tested were then excised and immunoblotted separately with monoclonal antibody 34C (J. Airey and J. Sutko, DSHB (Developmental Study Hybridoma Bank), University of Iowa, Iowa City, IA, U.S.A.), which recognizes both RyR1 and RyR3, monoclonal anti-calsequestrin-1 (MA3-913, Thermo Scientific), monoclonal anti-SERCA1 (MA3-911,Thermo Scientific) or polyclonal anti-GAPDH (glyceraldehyde-3-phosphate dehydrogenase) (FL-335 from Santa Cruz Biotechnology) antibodies. Membranes were then incubated with either goat anti-mouse or goat anti-rabbit horseradish-peroxidase-conjugated secondary antibody and developed with SuperSignal ultra chemiluminescent substrate (Pierce) and the intensity of the signal was collected using a Kodak Imaging Station 4000MM PRO (Carestream Health). Band densitometry of the identified proteins was performed using Kodak MI Software (version 4.5.1 ES). For each blot net band intensities were normalized to GAPDH expression to correct for protein loading and expressed as the fraction of the corresponding band of RyR1-expressing myotubes.

Data analysis

Statistical differences among datasets were evaluated using one-way Kruskal–Wallace ANOVA (non-parametric) analysis using Prism 5.0 (GraphPad Software). Unless indicated otherwise, results are means $\pm$ S.D.

[1] These authors contributed equally to this work.
[2] Correspondence may be addressed to either of these authors (email jenny@gsu.edu or cperez@zeus.bwh.harvard.edu).

© 2014 The Author(s)
The author(s) has paid for this article to be freely available under the terms of the Creative Commons Attribution Licence (CC-BY) (http://creativecommons.org/licenses/by/3.0/) which permits unrestricted use, distribution and reproduction in any medium, provided the original work is properly cited.

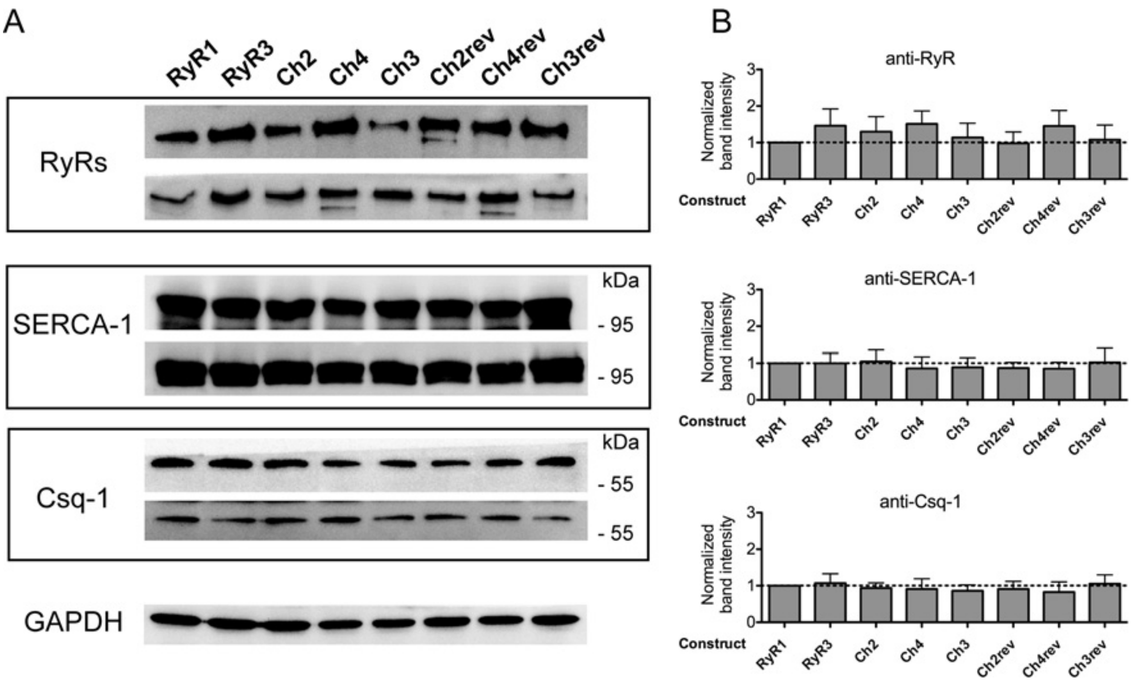

Figure S1 Relative expression of chimaeric RyRs in dyspedic myotubes

(**A**) Western blot analysis of crude membrane homogenates showing relative expression levels of RyRs, SERCA1 and calsequestrin-1 (Csq-1) of dyspedic myotubes infected with the wild-type or chimaeric constructs shown in Figure 1(A) of the main text. For each immunoblot, representative results of two different membrane homogenates are shown to highlight intrinsic variability between preparations. (**B**) Mean ± S.D. band intensity for each protein expressed as a fraction of the RyR1-expressing lane (broken line) for four to five blots from two separated membrane preparations. ANOVA one-way analysis of variance (Tukey's multiple comparison test) detected not statistically significant differences in band intensity among the different constructs.

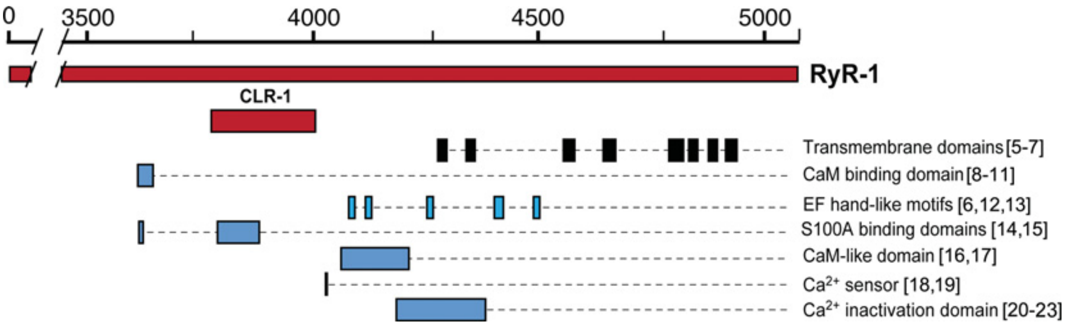

Figure S2 Localization of Ca^{2+}-binding/regulatory regions of RyR1

Mapping of the CLR-1 region within the context of the C-terminal tail of RyR1. Boxes represent the location of various domains of RyR1 reported to be involved in Ca^{2+}-binding or Ca^{2+}-mediated regulation. The location of CLR-1 does not appear to coincide with any of the prospective Ca^{2+}-binding regions currently identified in RyR1.

REFERENCES

1 Maniccia, A. W., Yang, W., Li, S. Y., Johnson, J. A. and Yang, J. J. (2006) Using protein design to dissect the effect of charged residues on metal binding and protein stability. Biochemistry **45**, 5848–5856 CrossRef PubMed

2 Zhou, Y., Xue, S., Chen, Y. and Yang, J. J. (2013) Probing Ca^{2+}-binding capability of viral proteins with the EF-hand motif by grafting approach. Methods Mol. Biol. **963**, 37–53 CrossRef PubMed

3 Laemmli, U. K. (1970) Cleavage of structural proteins during the assembly of the head of bacteriophage T4. Nature **227**, 680–685 CrossRef PubMed

4 King, Jr, L. E. and Morrison, M. (1976) The visualization of human erythrocyte membrane proteins and glycoproteins in SDS polyacrylamide gels employing a single staining procedure. Anal. Biochem. **71**, 223–230 CrossRef PubMed

5 Grunwald, R. and Meissner, G. (1995) Lumenal sites and C terminus accessibility of the skeletal muscle calcium release channel (ryanodine receptor). J. Biol. Chem. **270**, 11338–11347 CrossRef PubMed

6 Takeshima, H., Nishimura, S., Matsumoto, T., Ishida, H., Kangawa, K., Minamino, N., Matsuo, H., Ueda, M., Hanaoka, M., Hirose, T. et al. (1989) Primary structure and expression from complementary DNA of skeletal muscle ryanodine receptor. Nature **339**, 439–445 CrossRef PubMed

7 Zorzato, F., Fujii, J., Otsu, K., Phillips, M., Green, N. M., Lai, F. A., Meissner, G. and MacLennan, D. H. (1990) Molecular cloning of cDNA encoding human and rabbit forms of the Ca^{2+} release channel (ryanodine receptor) of skeletal muscle sarcoplasmic reticulum. J. Biol. Chem. **265**, 2244–2256 PubMed

8 Chen, S. R. and MacLennan, D. H. (1994) Identification of calmodulin-, Ca^{2+}-, and ruthenium red-binding domains in the Ca2 + release channel (ryanodine receptor) of rabbit skeletal muscle sarcoplasmic reticulum. J. Biol. Chem. **269**, 22698–22704 PubMed

9 Menegazzi, P., Larini, F., Treves, S., Guerrini, R., Quadroni, M. and Zorzato, F. (1994) Identification and characterization of three calmodulin binding sites of the skeletal muscle ryanodine receptor. Biochemistry **33**, 9078–9084 CrossRef PubMed

© 2014 The Author(s)
The author(s) has paid for this article to be freely available under the terms of the Creative Commons Attribution Licence (CC-BY) (http://creativecommons.org/licenses/by/3.0/) which permits unrestricted use, distribution and reproduction in any medium, provided the original work is properly cited.

10 Rodney, G. G., Krol, J., Williams, B., Beckingham, K. and Hamilton, S. L. (2001) The carboxy-terminal calcium binding sites of calmodulin control calmodulin's switch from an activator to an inhibitor of RYR1. Biochemistry **40**, 12430–12435 CrossRef PubMed

11 Yamaguchi, N., Xin, C. and Meissner, G. (2001) Identification of apocalmodulin and Ca^{2+}-calmodulin regulatory domain in skeletal muscle Ca^{2+} release channel, ryanodine receptor. J. Biol. Chem. **276**, 22579–22585 CrossRef PubMed

12 Hamada, T., Sakube, Y., Ahnn, J., Kim, D. H. and Kagawa, H. (2002) Molecular dissection, tissue localization and Ca^{2+} binding of the ryanodine receptor of *Caenorhabditis elegans*. J. Mol. Biol. **324**, 123–135 CrossRef PubMed

13 Xiong, H., Feng, X., Gao, L., Xu, L., Pasek, D. A., Seok, J. H. and Meissner, G. (1998) Identification of a two EF-hand Ca^{2+} binding domain in lobster skeletal muscle ryanodine receptor/Ca^{2+} release channel. Biochemistry **37**, 4804–4814 CrossRef PubMed

14 Treves, S., Scutari, E., Robert, M., Groh, S., Ottolia, M., Prestipino, G., Ronjat, M. and Zorzato, F. (1997) Interaction of S100A1 with the Ca^{2+} release channel (ryanodine receptor) of skeletal muscle. Biochemistry **36**, 11496–11503 CrossRef PubMed

15 Wright, N. T., Prosser, B. L., Varney, K. M., Zimmer, D. B., Schneider, M. F. and Weber, D. J. (2008) S100A1 and calmodulin compete for the same binding site on ryanodine receptor. J. Biol. Chem. **283**, 26676–26683 CrossRef PubMed

16 Gangopadhyay, J. P. and Ikemoto, N. (2006) Role of the Met^{3534}–Ala^{4271} region of the ryanodine receptor in the regulation of Ca^{2+} release induced by calmodulin binding domain peptide. Biophys. J. **90**, 2015–2026 CrossRef PubMed

17 Xiong, L., Zhang, J. Z., He, R. and Hamilton, S. L. (2006) A Ca^{2+}-binding domain in RyR1 that interacts with the calmodulin binding site and modulates channel activity. Biophys. J. **90**, 173–182 CrossRef PubMed

18 Chen, S. R., Ebisawa, K., Li, X. and Zhang, L. (1998) Molecular identification of the ryanodine receptor Ca^{2+} sensor. J. Biol. Chem. **273**, 14675–14678 CrossRef PubMed

19 Li, P. and Chen, S. R. (2001) Molecular basis of Ca^{2+} activation of the mouse cardiac Ca^{2+} release channel (ryanodine receptor). J. Gen. Physiol. **118**, 33–44 CrossRef PubMed

20 Bhat, M. B., Zhao, J., Takeshima, H. and Ma, J. (1997) Functional calcium release channel formed by the carboxyl-terminal portion of ryanodine receptor. Biophys. J. **73**, 1329–1336 CrossRef PubMed

21 Chen, S. R., Zhang, L. and MacLennan, D. H. (1992) Characterization of a Ca^{2+} binding and regulatory site in the Ca^{2+} release channel (ryanodine receptor) of rabbit skeletal muscle sarcoplasmic reticulum. J. Biol. Chem. **267**, 23318–23326 PubMed

22 Du, G. G., Khanna, V. K. and MacLennan, D. H. (2000) Mutation of divergent region 1 alters caffeine and Ca^{2+} sensitivity of the skeletal muscle Ca^{2+} release channel (ryanodine receptor). J. Biol. Chem. **275**, 11778–11783 CrossRef PubMed

23 Du, G. G. and MacLennan, D. H. (1999) Ca^{2+} inactivation sites are located in the COOH-terminal quarter of recombinant rabbit skeletal muscle Ca^{2+} release channels (ryanodine receptors). J. Biol. Chem. **274**, 26120–26126 CrossRef PubMed

Received 25 November 2013/3 March 2014; accepted 18 March 2014
Published as BJ Immediate Publication 18 March 2014, doi:10.1042/BJ20131553

© 2014 The Author(s)
The author(s) has paid for this article to be freely available under the terms of the Creative Commons Attribution Licence (CC-BY) (http://creativecommons.org/licenses/by/3.0/) which permits unrestricted use, distribution and reproduction in any medium, provided the original work is properly cited.
